# Novel Antimicrobial Peptides with High Anticancer Activity and Selectivity

**DOI:** 10.1371/journal.pone.0126390

**Published:** 2015-05-13

**Authors:** Hung-Lun Chu, Bak-Sau Yip, Kuan-Hao Chen, Hui-Yuan Yu, Ya-Han Chih, Hsi-Tsung Cheng, Yu-Ting Chou, Jya-Wei Cheng

**Affiliations:** 1 Institute of Biotechnology and Department of Medical Science, National Tsing Hua University, Hsinchu, 300, Taiwan; 2 Department of Neurology, National Taiwan University Hospital Hsinchu Branch, Hsinchu, 300, Taiwan; Nanyang Technological University, SINGAPORE

## Abstract

We describe a strategy to boost anticancer activity and reduce normal cell toxicity of short antimicrobial peptides by adding positive charge amino acids and non-nature bulky amino acid β-naphthylalanine residues to their termini. Among the designed peptides, K4R2-Nal2-S1 displayed better salt resistance and less toxicity to hRBCs and human fibroblast than Nal2-S1 and K6-Nal2-S1. Fluorescence microscopic studies indicated that the FITC-labeled K4R2-Nal2-S1 preferentially binds cancer cells and causes apoptotic cell death. Moreover, a significant inhibition in human lung tumor growth was observed in the xenograft mice treated with K4R2-Nal2-S1. Our strategy provides new opportunities in the development of highly effective and selective antimicrobial and anticancer peptide-based therapeutics.

## Introduction

The development of cationic antimicrobial peptides (CAPs) as functional therapeutics to fight against infectious diseases and cancer has become an important area [1–3]. Cationic antimicrobial peptides, important for regulating the innate immune system of plants, insects, and animals [4], are recognized as candidates against bacteria and fungi originally [5, 6]. CAPs are normally characterized by their positive charges and amphipathic features, which enable them to bind to negatively charged bacterial cell membranes and cause the disruption of the membrane, hence the death of bacteria [7, 8]. The membrane lytic property of CAPs makes them potential therapeutics for overcoming the antibiotic resistance [6].

Although tremendous efforts have been put into the development of new treatments, cancer remains the major cause of death [9]. Chemotherapies, despite their severe side effects to normal cells and tissues, and the easy formation of multi-drug resistances, are still the principal drugs used to treat cancer in the advanced or metastatic stages [9]. Thus, the development of new cancer drugs with low toxicity to normal cells and a new mode of mechanism that can avoid multi-drug resistance may provide a new direction for anticancer therapy. The outer membranes of cancer cells have been reported to carry more negatively charged molecules, such as phosphatidylserines, negative glycoproteins, and glycosaminoglicans, than normal cells [10–12]. Owing to their cationic and amphipathic features, CAPs may bind to cancer cells by electrostatic interactions, and hence lead to cytotoxicity of cancer cells with either necrosis or apoptosis phenotype [13–18]. Indeed, several CAPs have been recognized as novel cancer-targeted therapeutics with better solubility and lower cost [19]. Although many CAPs are discovered to have effective anticancer activity, there are several challenges, such as salt sensitivity in physiological conditions, high toxicity to normal cells, and susceptibility to proteolytic digestion, constraining their further applications [19]. Currently, several methods have been reported to overcome these problems, including D-form amino acid substitution [20–22], fusion with functional peptides [22, 23], and conjugation with chemotherapeutic agents [24, 25].

PEM-2-W5K/A9W (Ac-KKWRKWLKWLAKK-NH_2_) is an effective antimicrobial peptide against bacteria and fungi under physiological ionic concentration [6, 26]. S1 (Ac-KKWRKWLAKK-NH_2_) is a shorter version of PEM-2-W5K/A9W with similar antibacterial and antifungal activities but possessing less hemolytic activities. However, unlike PEM-2-W5K/A9W, S1 loses its antimicrobial activities dramatically under physiological ionic concentration [27]. Previously, we have developed a strategy to boost activities and salt resistance of short antimicrobial peptides by modulating their lipophilicity with the addition of non-natural bulky amino acid such as β-naphthylalanine (Nal) to their N- or C-terminus [27]. This strategy has been applied successfully to S1. In order to determine if this strategy can be applied to CAPs to enhance their anticancer activity, herein, we have designed a series of Nal-embedded S1 analogs and evaluated their effects on antimicrobial and anticancer activities.

## Materials and Methods

### Ethics statement

Human venous blood was collected from three healthy volunteers with prior written informed consent and approval from the Institution Review Broad of the National Taiwan University Hospital Hsin-chu Branch.

All animal experiments were performed in accordance with the animal guidelines of the National Tsing Hua University Institutional Animal Care and Use Committee (Permit Number: 10260). All nude mice were sacrificed under CO_2_, and all efforts were made to minimize suffering.

### Peptide preparation

All peptides were purchased from Kelowna Int’l Scientific Inc. (Taiwan). The identity of the peptides was checked by electrospray mass spectroscopy and the purity (>95%) was assessed by HPLC. Peptide concentration was determined by using the UV/Visible spectrophotometer at 280 nm. Buffers were prepared in double glass-distilled water.

### Bacteria culture

*Escherichia coli* strain (ATCC 25922), *Staphylococcus aureus* subsp strain (ATCC 25923, methicillin-resistant), and *Pseudomonas aeruginosa* Migula strain (ATCC 27853, ampicillin-resistant) were used to test the antibacterial activity of the peptides. Bacteria were cultured in sterilized MH at 200 rpm and 37°C for 8 hours. After 8 hours culture, concentration of the inoculums were determined by measuring absorbance of optical density at 600 nm (OD 600 = 1, equal to approximately 10^8^ CFU/mL) with UV/Visible spectrophotometer.

### Antimicrobial activity

The antibacterial activities were determined by the standard broth microdilution method of National Committee for Clinical Laboratory Standards with the LYM broth. The LYM broth contains 5.4 mM KCl, 5.6 mM Na_2_HPO_4_, 0.5 mM MgSO_4_, and 1.0 mM sodium citrate. In addition, 0.4 mg of ZnCl_2_, 2.0 mg of FeCl_3_·6H_2_O, 0.1 mg of CuSO_4_·5H_2_O, 0.1 mg of MnSO_4_·H_2_O, 0.1 mg of Na_2_B_4_O_7_·10H_2_O, 700 mg of amino acid mixtures without tryptophan (Clontech), and 20 mg of L-Tryptophan were added per liter of medium. A vitamin mixture (100X, Sigma) and glucose at final concentration of 2% were also added. We made 1 μl peptide solutions (ranging from 5000μg/ml to 78μg/ml in serial dilution) and mixed with 99 μl inoculum (5 x 10^5^ CFU/ml) in polypropylene 96-well plate. We measured the turbidity at OD 600 nm by ELISA plate reader (Thermo Max, Molecular Devices, Sunnyvale, CA). The absorbance of culture medium and inoculum suspension without peptides were used as the negative and positive control, respectively. The MIC value is the lowest concentration of peptide at which there is no obvious growth (equal or more than 90%). MICs were converted to a color scale and displayed using the TreeView Program [28, 29]. All peptides were tested in triplicate.

### Hemolytic activity

Human venous blood was collected by a venous blood collection tube (BD Vacutainer, REF 367525). Serum was removed by PBS buffer washing and centrifugation at 800 g for 5 min. The above processes were repeated at least three times to remove the serum completely. 50 μl of peptides (ranging 1.6 mM to 3.1 μM in serial dilution) mixed with 50 μl of 10% hRBC and incubated at 37°C for 1 hour. The supernatant were collected after centrifugation at 800 g for 5 min. The amount of hemoglobin released from hRBC was determined by measuring the absorbance at 405 nm. 10% hRBC without peptide and treated with 1% Triton X-100 represented negative and positive control, respectively.

### Cell culture

Human lung cancer line PC9 and A549, oral squamous cell carcinoma cell line OECM-1 were cultured in RPMI medium supplemented with 10% fetal bovine serum and antibiotic. Human tongue carcinoma cell line SAS, oral cancer cell line C9, and human diploid fibroblast (HFW) were cultured in DMEM medium supplemented with 10% fetal bovine serum and antibiotic. Cells were cultured in a humidified incubator containing 5% CO_2_ at 37°C. PC9 and the gefitinib resistant PC9 strain (PC9-G) were received from Dr. Yu-Ting Chou, Institute of Biotechnology, National Tsing Hua University. The original source of PC9 lung cancer cell line was kindly provided by Dr. Cheng-Wen Wu in Academia Sinica, Taiwan [30]. PC9-G was generated from culturing PC9 cells in gefitinib (500 nM) for 60 days.

### Cell toxicity

The MTT assay was employed to determine the *in vitro* cytotoxicity. All cancer cell lines were seeded in 96-well plate with concentration 5000 cells/100 μl/well and incubated for 24 hrs. HFW was seeded with 8000 cells/100 μl/well. After medium was removed, 100 μl fresh medium containing peptide (ranging from 50 μM to 3.13 μM, HFW was treated with 75 μM and 100 μM peptide additionally) was added to the wells. Following 24 h incubation, fresh medium with MTT (0.5 mg/ml) was replaced and incubated for 3 h. After medium/MTT was removed, DMSO was added at 100 μl for dissolving the formazan crystal. Cell survival rate was calculated by measuring the absorbance at 540 nm using Multi-labeled Microplate Reader (VICTOR3). Medium without peptide and mixed with H_2_O_2(aq)_ represented positive and negative control, respectively.

### Cell live image

PC9 and HFW cells (~10^5^ cells) were pre-seeded in 6-cm polystyrene dishes for 24 h. Cell nuclear is labeled by 4',6-diamidino-2-phenylindole (DAPI) with final concentration of 10 μg/ml. After 10min incubation, the cells were washed by PBS. FITC-K4R2Nal2S1-NH_2_ was added to the dishes with the final concentration of 12 μM. After incubation for 5, 10, 20 min or 1 h at 37°C, the cells were washed with PBS. The images of FITC-K4R2Nal2S1-NH_2_ in PC9 cells and HFW cells were observed using the Inverted Fluorescent Microscope Zeiss/ Observer.Z1.

### Western blotting

PC9 cells were seeded in 10-cm polystyrene dishes for 48 h. About seventy percent full of cells were treated with 12 μM K4R2Nal2S1 for 10 mins, 1 h or 24 h. RPMI medium without peptide treated for 24 h which was used as negative control. Cells were collected by 200 μl RIPA and protease inhibitor blended buffer. After sonication and centrifugation for 10 mins, 13000 rpm at 4°C, supernatants (lysis of cell) were collected. Protein concentrations of cell extracts were determined by Bradford reagent. Equal amount of boiled lysates (total 50μg) were separated on 10% acrylamide gel. The gel was transferred to PVDF membrane in electro-blot system, 300 V, 350 A, and 80 mins. The membrane was incubated in blocking buffer (5% skim milk, TBST buffer) for 1 h in room temperature and washed in TBST buffer twice. Blocked membrane was incubated with Caspase-3 antibody (EPITOMICS, clone ID:E83-77) overnight at 4°C, washed for five times, and then incubated in secondary antibody (HRP, GeneTex catalogue number: GTX21311-01) for 1 h in room temperature. The signals were visualized by enhanced chemiluminescence (ECL) and recorded by a detected system (ImageQuant LAS 4000 mini).

### Mice and pathological studies

12 male nude mice (BALB/cAnN.Cg-Foxn1nu/CrlNarl) were purchased from National Laboratory Animal Center, Taiwan. 100 μl human lung cancer cell PC9 (3 x 10^6^ cells) in Matrigel (Corning) was injected subcutaneously into the dorsal side of 5-week-old male nude mice. Each mouse was inoculated two sites on its back [31]. 5 days after implantation (cancer size>95mm^3^), 12 mice were allocated randomly into two group. One group was received K4R2Nal2S1 (5mg/Kg, dissolving in 100μl PBS buffer) by tail vein injection three times a week, the other group was injected PBS as control. Body weight and cancer size were measured three times a week. The cancer volume was calculated by formula of width^2^×length×0.52. The cancer volume below 100% of the pretreatment volume was defined as “cancer reduction” [31]. “Cancer reduction” was confirmed when the mice were dissected. The mice were sacrificed after treatment of 40 days, the cancers were removed, photographed, and weighed. All animal experiments were performed in accordance with the animal guidelines of the National Tsing Hua University Institutional Animal Care and approved by Animal Care Committee.

Solid cancers were fixed in 4% formaldehyde buffer. Paraffin-embedded tissues were cut to 2 μm-thickness sections, and deparaffinized in ultraclear buffer (J.T. Baker) and graded ethanol. The morphology of cancers was obtained by H&E stained sections. In addition, the sections were immunostained with anti-cleaved-PARP (1:100) antibody (Cell Signaling, clone number: D65E10). Tissue images were captured using light microscope (Eclipse E400, Nikon) with digital microscopy camera (AxioCam ICc 5, ZEISS), at 40X, 200X and 400X fields.

## Results

### Peptide design

Previously, we have developed a strategy to boost salt resistance and serum stability of short antimicrobial peptides by adding β-naphthylalanine to their termini [27]. This strategy has been applied successfully to S1 and the ultrashort peptide KWWK. However, peptides with β-naphthylalanine end-tags also demonstrate higher cell lytic activity (cytotoxicity) [27]. This problem may be compensated by adding positive charge residues to N- and/or C-terminus of the antimicrobial peptides [32]. Herein, we have designed and synthesized Nal2-S1, K4R2-Nal2-S1, and K6-Nal2-S1 and compared their antimicrobial and anticancer activities as well as their cytotoxicities with the parent peptide S1. The sequences and molecular weights of S1, Nal2S1, K4R2-Nal2-S1, and K6-Nal2-S1 are listed in Table 1.

**Table 1:** Primary structure of S1 and its analogues.

Peptide	Sequence^a^	Molecular Weight (Da)
S1	Ac-KKWRKWLAKK-NH_2_	1412.79
Nal2-S1	Ac-Nal-Nal-KKWRKWLAKK-NH_2_	1807.2
K4R2-Nal2-S1	Ac-KKKKRR-Nal-Nal-KKWRKWLAKK-NH_2_	2631.58
K6-Nal2-S1	Ac-KKKKKK-Nal-Nal-KKWRKWLAKK-NH_2_	2576.26

^a^ Nal = β-naphthylalanine

### Antimicrobial activity

**Fig 1:**
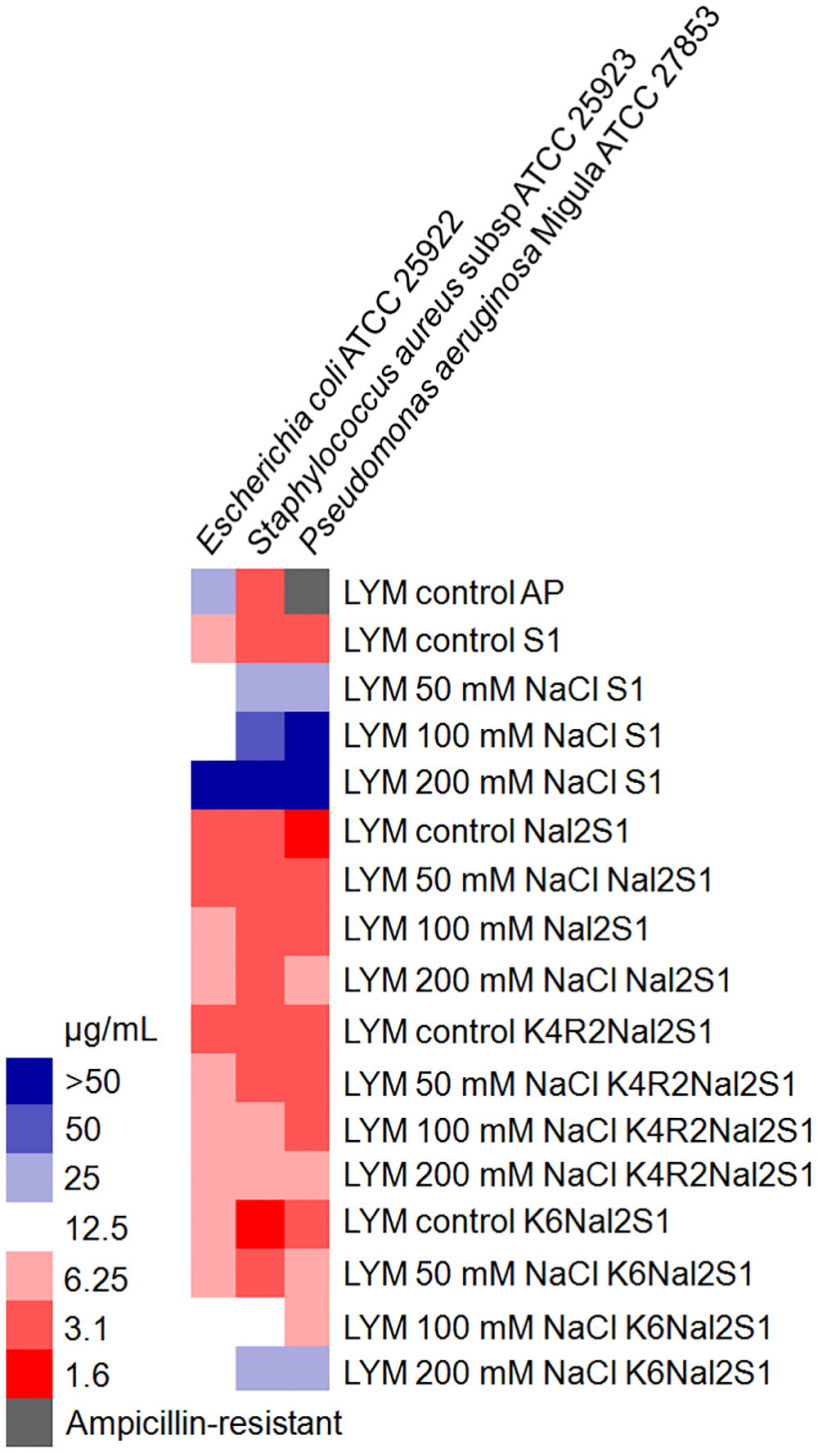
MIC values displayed on a color scale for Ampicillin (AP), S1, Nal2-S1, K4R2-Nal2-S1, and K6-Nal2-S1 under different concentrations of NaCl.

The activities of S1 and its analogs were tested against gram-positive and gram-negative bacteria under several salt concentrations. The MICs of the peptides were analyzed, showing that all three peptides are very effective against bacteria in LYM broth condition (Fig 1). Nal2-S1 and K4R2-Nal2-S1 demonstrate promising activities in high-salt conditions. However, the activities of K6-Nal2-S1 are diminished by the addition of 100 or 200 mM NaCl.

### Cytotoxicity

**Fig 2:**
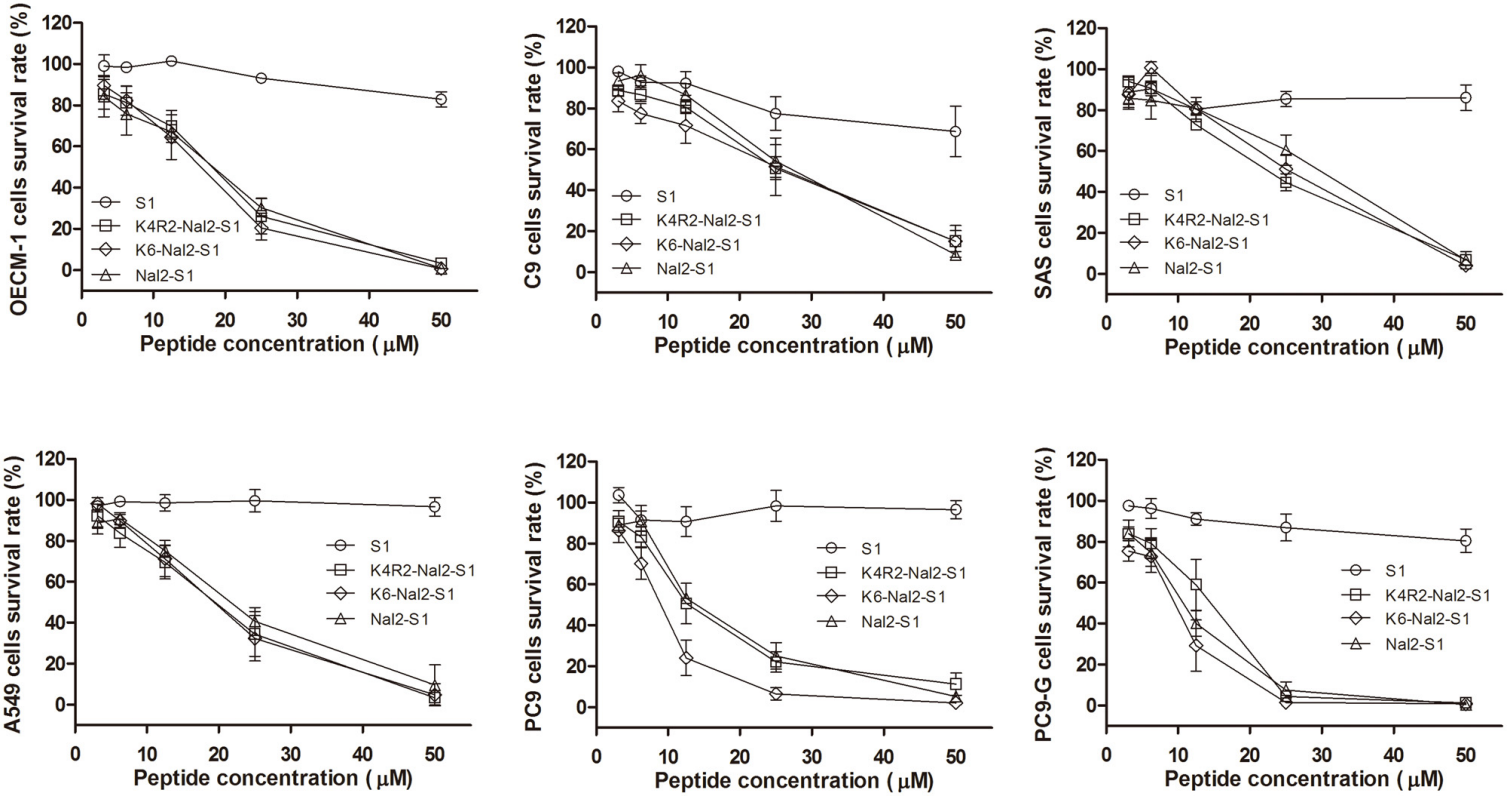
Plots of the activities of S1, Nal2-S1, K4R2-Nal2-S1, and K6-Nal2-S1 against OECM-1, C9, SAS, A549, PC9, PC9-G cancer cell lines.

The cytotoxicities of S1 and its analogs on human lung cancer cells (i.e, PC9, PC9-G and A549), human oral cancer cells (i.e, C9, OECM-1, and SAS), and human fibroblast (HFW) were evaluated by MTT assay for 24 hours. The data showed that the three Nal-embedded peptides all have potent anticancer activities against different cancer cell lines (Fig 2). Similar results were observed at earlier time points (i.e. 3 hours and 12 hours) (S1 and S2 Figs).

**Fig 3:**
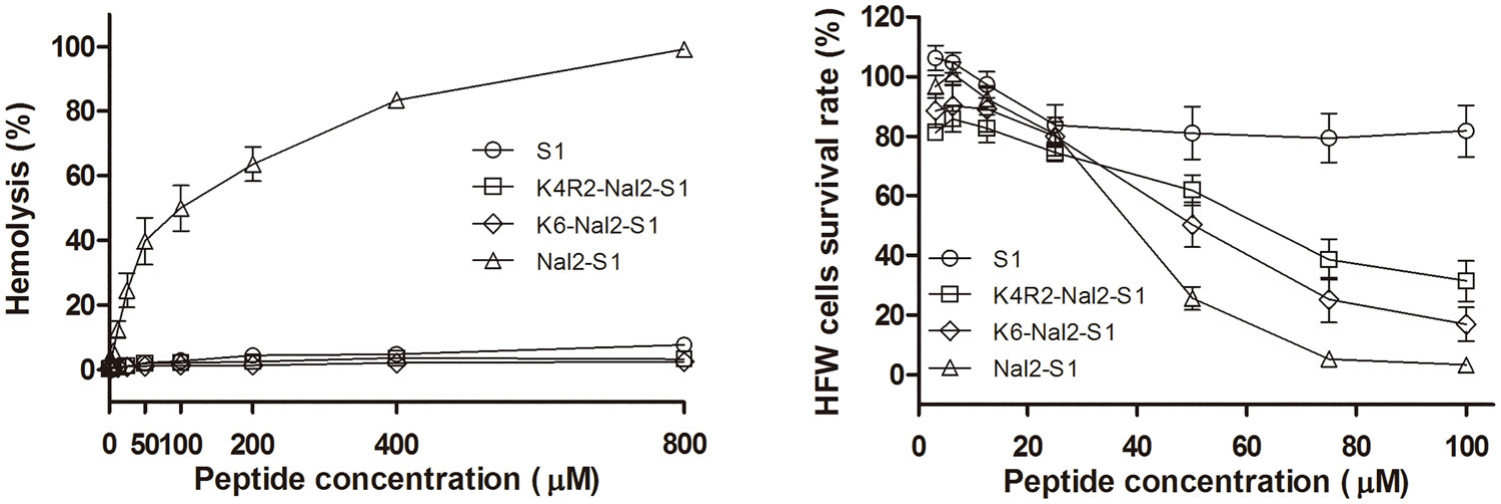
Hemolysis and cytotoxicity to human red blood cells (hRBCs) and human fibroblast (HFW) cells of S1, Nal2-S1, K4R2-Nal2-S1, and K6-Nal2-S1.

The selectivity of the peptides was investigated using human red blood cells (hRBCs) and human fibroblast (HFW) (Fig 3). The lytic activity of all peptides toward hRBCs was tested at 37°C for 1 h incubation and calculated by minimal hemolytic concentration. Nal2-S1 displayed 10% hemolytic activity at 25 μM peptide concentration. Surprisingly, the hemolytic activity of K4R2-Nal2-S1 and K6-Nal2-S1 were not found even at 800 μM. The degrees of cytotoxicities of these peptides to HFW were found to be S1 < K4R2-Nal2-S1 < K6-Nal2-S1 < Nal2-S1. As K4R2-Nal2-S1 displayed better salt resistance and less toxic to hRBCs and human fibroblast than Nal2-S1 and K6-Nal2-S1, K4R2-Nal2-S1 was selected to investigate its anticancer activities in PC9 cancer cell line and xenograft animal model.

### *In vitro* anticancer mechanism

**Fig 4:**
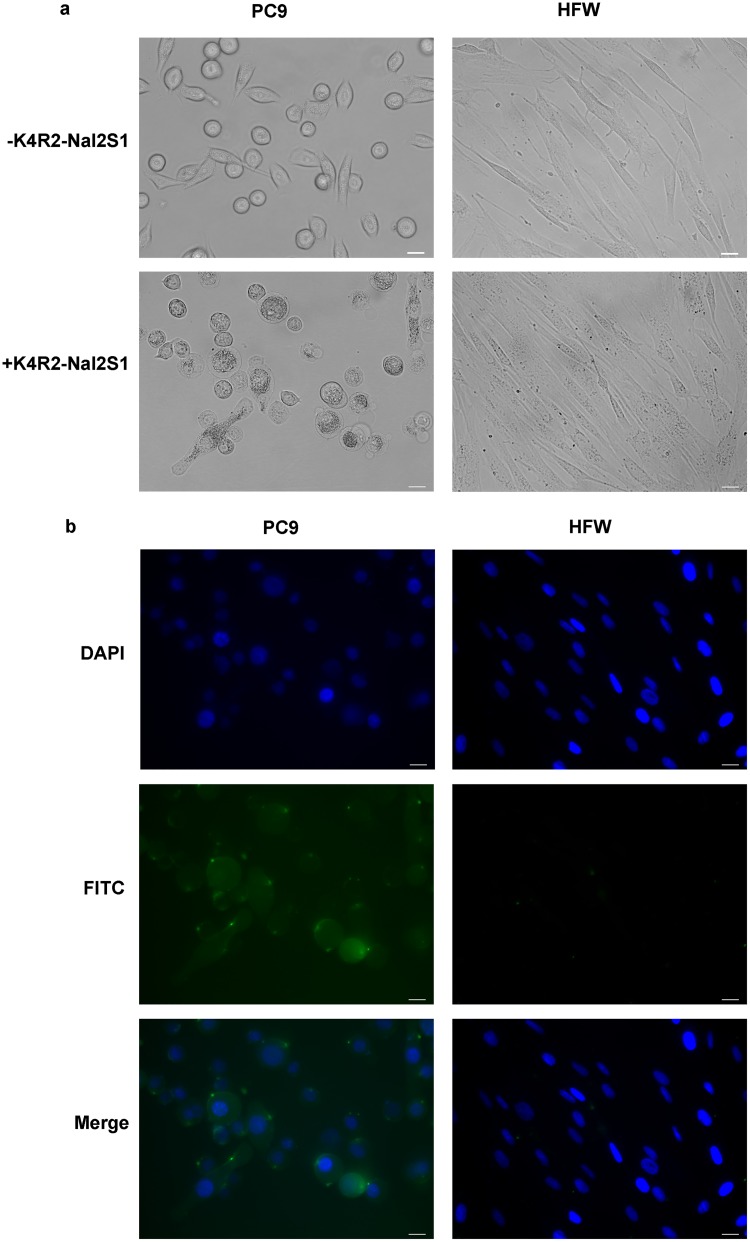
K4R2-Nal2-S1 binds cell membrane and induces apoptosis in cancer cells. (a) Representative phase-contrast images of PC9 and HFW cells treated with or without 12 μM FITC-K4R2-Nal2-S1 for 5 min. (b) Immunofluorescence analysis for interaction of FITC-conjugated K4R2-Nal2-S1 with PC9 and HFW cells. Cells were prestained with DAPI for nuclear detection, followed by FITC-K4R2-Nal2-S1 (12 μM) treatment for 1 h. DAPI and FITC were presented in blue and green signals under UV and blue light sources, respectively. All Scale bars = 20 μm.

**Fig 5:**
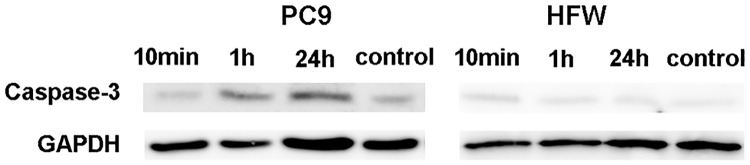
Western blot analysis for activated caspase 3 expression to monitor cellular apoptosis in PC9 and HFW cells at the indicated time points. GAPDH served as a loading control.

To investigate the mode of actions of K4R2Nal2-S1 on human cancer cell line (PC9) and human fibroblast (HFW), cells were treated with FITC-labeled K4R2-Nal2-S1. Nucleus was labeled with DAPI, and the blue signal was observed by UV exciting light. The fluorescence distribution of FITC-labeled K4R2-Nal2-S1 on cell membrane was visualized by the inverted fluorescent microscope. Phase-contrast microscopy showed that K4R2-Nal2-S1 treatment induced cellular swelling in PC9 but not in HFW cells (Fig 4a). Moreover, immunofluorescence analysis revealed that FITC-labeled K4R2-Nal2-S1 treatment caused puncta formation on cell membrane in PC9, but not in HFW cells (Fig 4b). Immunoblotting indicated that K4R2-Nal2-S1 treatment activated caspase 3 in PC9 but not in HFW cells, suggesting the involvement of apoptosis in K4R2-Nal2-S1 mediated cell death (Fig 5). Our data suggest that K4R2-Nal2-S1 preferentially binds cancer cells, causing apoptotic cell death.

### Inhibiting lung cancer growth in xenograft mouse model

**Fig 6:**
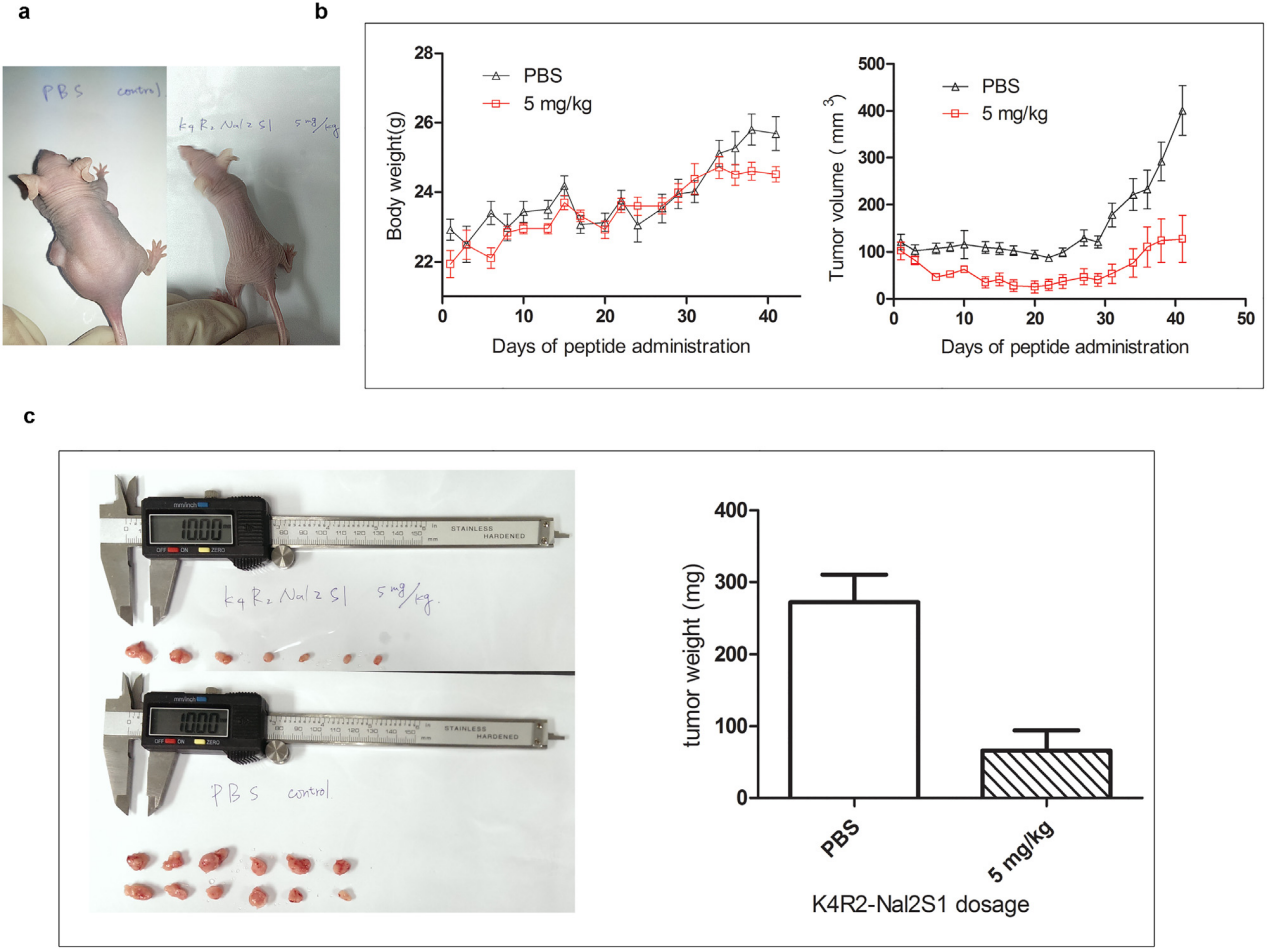
K4R2-Nal2-S1 treatment attenuates xenograft tumor growth. (a) Dorsal sides of male nude mice s.c. injected with PC9 human lung cancer cells and i.v. treated with K4R2Nal2-S1 (right) or PBS control (left) at the 46^th^ day after cancer cell implantation (5 days for tumor growth, 40 days for treatment, photographed on the 46^th^ day). (b) Mice body weight (left) and tumor volume (right) from (a) were monitored over the time period as indicated. (c) Exposed tumors (mice were sacrificed at the 46^th^ day after cancer cell implantation) of mice treated with K4R2Nal2-S1 (upper) or PBS (lower). 12 exposed tumors were found in the PBS group (6 mice x 2 side implantation), but only 7 exposed tumors were found in the K4R2Nal2-S1 treatment group. Total tumor weight for both groups were measured and shown in right.

To evaluate the anticancer effect of K4R2Nal2-S1 *in vivo*, PC9 cells were implanted subcutaneously to nude mice (Fig 6a) and followed by K4R2-Nal2-S1 injection via the intravenous route, at a dose of 5 mg/kg, three times weekly (Fig 6b). During the administration, body weight loss was not found in K4R2Nal2-S1 treated group (Fig 6b); however, a significant inhibition in tumor growth was observed in the mice treated with K4R2-Nal2-S1 (Fig 6b). K4R2-Nal2-S1 treatment also decreased the volume and weight of tumors harvested 40 days after injection (Fig 6c).

**Fig 7:**
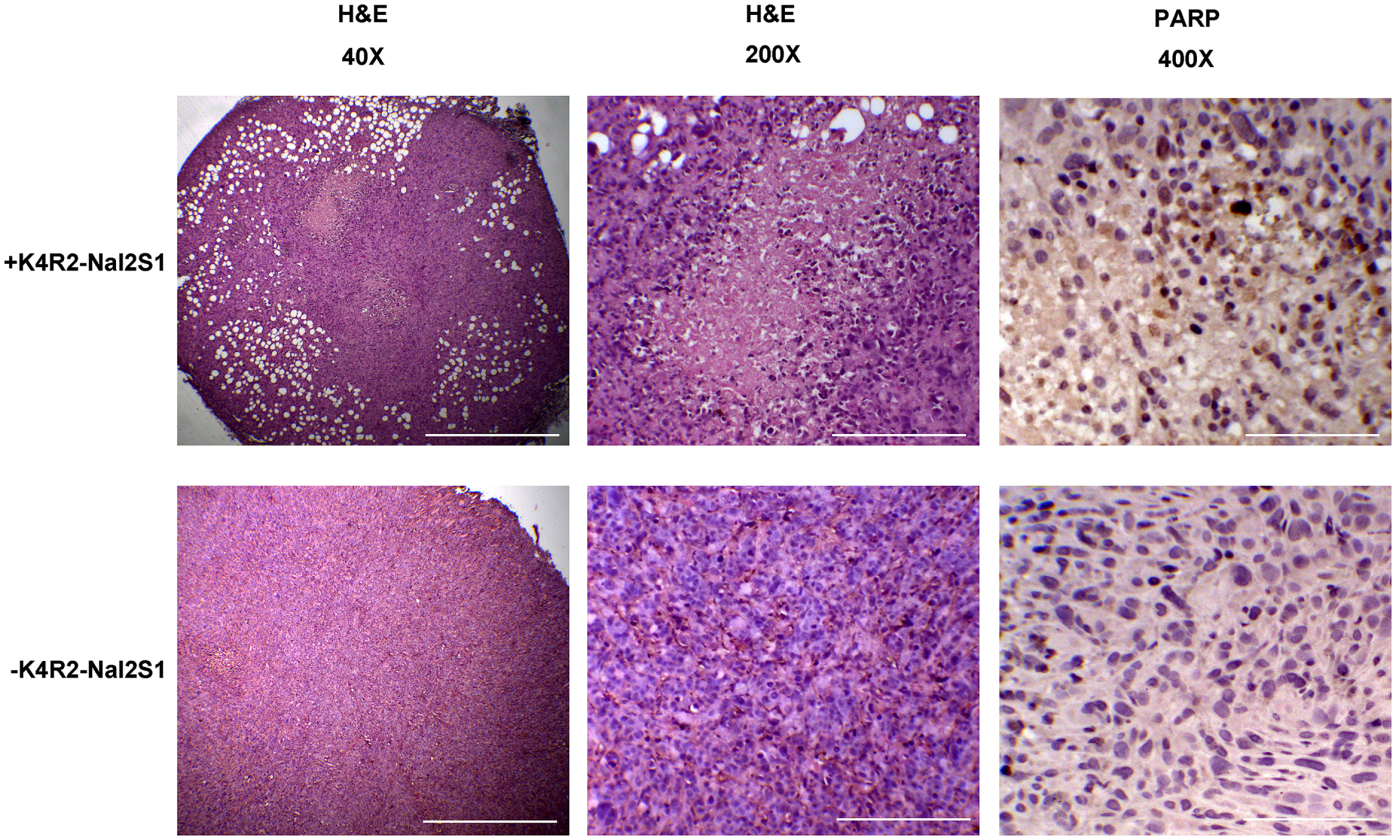
Excised xenografted tumors from the mice in Fig 3 were subjected to H&E staining and immunohistochemical assay for cleaved PARP expression to monitor cellular apopotosis. Scale bars = 500 μm, 100 μm, and 50 μm (40X, 200X, and 400X), respectively.

To examine the involvement of necrosis and apoptosis in K4R2Nal2-S1 mediated anticancer effect, lung tumors generated in the xenograft mouse model were excised and analyzed by H&E staining and immunohistochemistry for cleaved PARP, a marker for cell apoptosis. The H&E staining showed the presence of large scale of necrosis regions in K4R2-Nal2-S1 treated cancers but not in those treated with PBS (Fig 7). Immunohistochemistry identified increased expression of cleaved PARP next to the necrosis regions in tumors under K4R2-Nal2-S1 treatment. These data support the notion that K4R2Nal2-S1 treatment inhibits tumor growth.

## Discussion

Cationic antimicrobial peptides have been applied extensively to research in infectious diseases. For example, CAPs have been coated on calcium phosphate-coated titanium for the prevention of implant-associated infections [33, 34]. Also, a variety of hydrogels containing CAPs were developed for the prevention and treatment of infections after surgery [35–38]. On the other hand, only a few studies were reported on the development and application of CAPs in cancer treatment and most of these studies were focused on CAPs derived from natural amino acids [39–43]. Previously, we have discovered that the replacement of histidine or tryptophan residues, or the addition of non-natural bulky amino acid end-tags, such as β-naphthylalanine (Nal) and β-(4,4’-biphenyl)alanine (Bip), can enhance salt resistance and serum stability of short antimicrobial peptides [27, 44]. Based on the present study, the addition of β-naphthylalanine residues to S1, no matter at the N-terminus or embedded in the sequence, can help Nal2-S1, K4R2-Nal2-S1, and K6-Nal2-S1 to kill antibiotic resistant gram positive and negative bacterial strains under high salt conditions. The degree of salt resistance is Nal2-S1 = K4R2-Nal2-S1 > K6-Nal2-S1.

Hydrophobicity has been known as a key factor in the design and development of effective antimicrobial peptides. For example, the number and location of hydrophobic residues, as well as their hydrophobicity and type of hydrophobe are important factors that can affect the antimicrobial activities of amphipathic α-helical peptides [45, 46]. Also, the addition of tryptophan and/or phenylalanine end-tags, fatty acid, vitamin E, or cholesterol to the termini of antimicrobial peptides was shown to boost antimicrobial activities of short antimicrobial peptides [28, 47–53]. However, less has been addressed in the involvement of hydrophobicity in the development of anticancer antimicrobial peptides. The expression of negatively charged molecules such as phosphatidylserines, negative glycoproteins and glycosaminoglicans make cancer cell membranes similar to bacterial membranes. Unlike their parent peptide S1, Nal2-S1, K4R2-Nal2-S1, and K6-Nal2-S1 all exhibit effective activities against various human cancer cell lines. This may be due to the fact that the bulky β-naphthylalanine residues may help these peptides to penetrate deeper into the cancer cell membranes, hence making these peptides more efficient in disrupting the membranes.

Among Nal2-S1, K4R2-Nal2-S1, and K6-Nal2-S1, K4R2-Nal2-S1 demonstrates the best salt resistance, anticancer activity, less normal cell toxicity, and less hemolytic activity. The fluorescent microscopic results indicated that K4R2-Nal2-S1 binds to cancer cells and accumulates on the cell membranes. This is consistent with the hypothesis that the mechanism of anticancer activity of CAPs is similar to that of the antimicrobial activity. Moreover, different apoptotic hallmarks produced *in vitro* and *in vivo* indicate that the death of the peptide treated cancer cells arises from apoptosis. The mechanism of the anticancer activity of CAPs is thus different from the mechanism of other chemotherapeutic drugs.

To mimic the current route of administration (intravenous) used clinically for cancer treatments, K4R2-Nal2-S1 was injected intravenously and showed significant tumor growth inhibitory effects *in vivo* on xenografts in nude mice but interestingly without any toxicity such as enervation or body weight loss. Our data imply a feasible combination treatment with K4R2-Nal2-S1 and other chemotherapeutic drugs, with potential to improve therapeutic response and overcome the chemotherapy induced multi-drug resistances. Studies of this combination treatment are currently undergoing in our laboratory.

## Conclusions

Herein, we describe a strategy to boost anticancer activity and reduce normal cell toxicity of short antimicrobial peptides by adding positive charge amino acids and non-natural β-naphthylalanine residues to their N-termini. This strategy has been applied successfully to S1 as demonstrated in this study. In addition, we have demonstrated that one of the peptides designed, K4R2-Nal2-S1, is effective in reducing cancer burden in xenograft mouse model with no obvious toxicity to the hosts. Our results provide a potential therapeutic agent for future clinical applications.

## Supporting Information

S1 Fig Plots of the activities of S1, Nal2-S1, K4R2-Nal2-S1, and K6-Nal2-S1 against OECM-1, C9, SAS, A549, PC9, PC9-G cancer cell lines at 3 hrs. (TIF)

S2 Fig Plots of the activities of S1, Nal2-S1, K4R2-Nal2-S1, and K6-Nal2-S1 against OECM-1, C9, SAS, A549, PC9, PC9-G cancer cell lines at 12 hrs. (TIF)
