# Supplementary figures and images for: Novel Antimicrobial Peptides with High Anticancer Activity and Selectivity
Source: PLoS One. 2015 May 13;10(5):e0126390. doi: 10.1371/journal.pone.0126390 (PMC4430538; doi:10.1371/journal.pone.0126390)

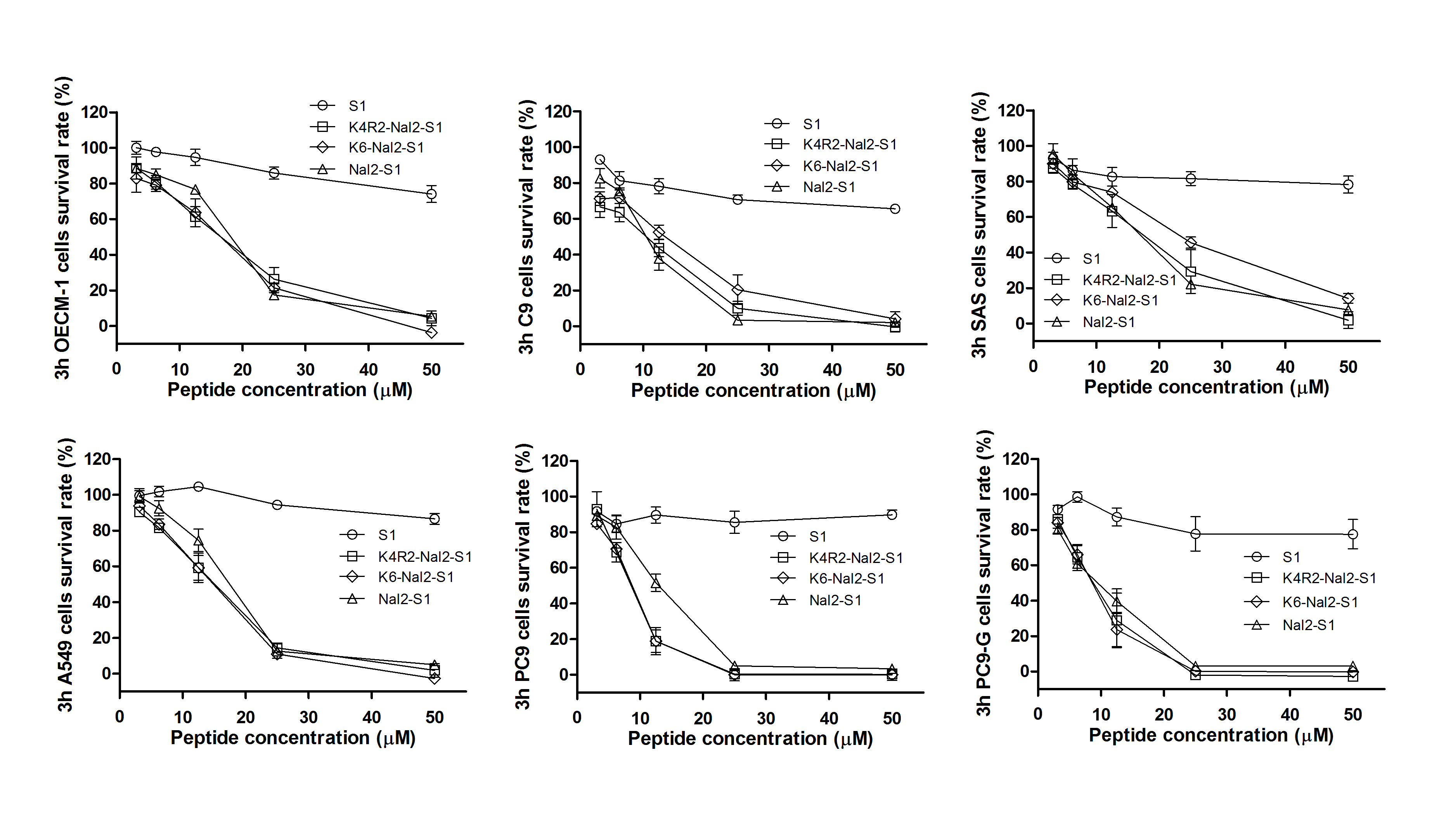

Supplement: S1 Fig — (TIF) [file pone.0126390.s001.tif]

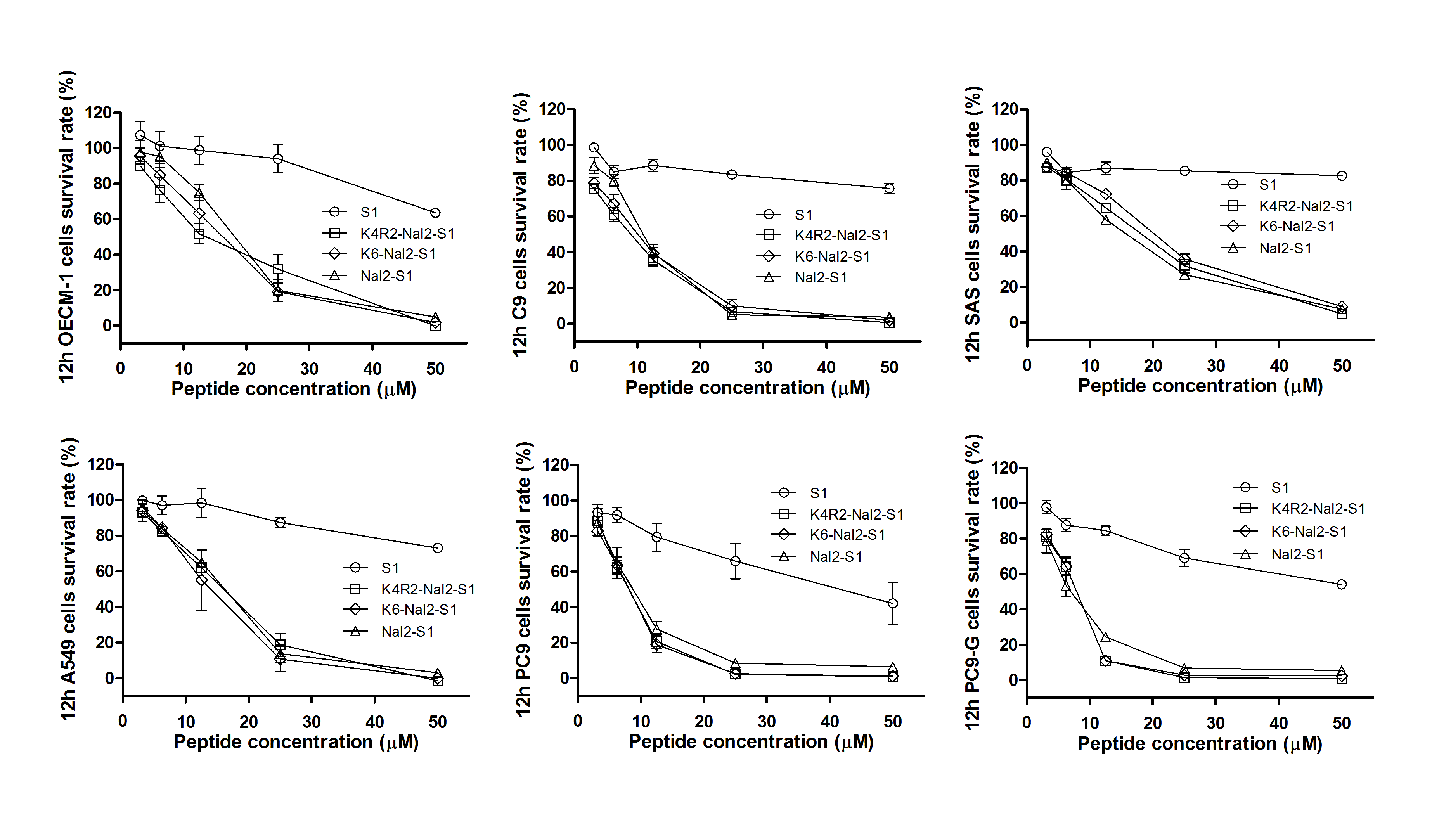

Supplement: S2 Fig — (TIF) [file pone.0126390.s002.tif]
